# SOCIAL DETERMINANTS OF HEALTH AND EMERGENCY DEPARTMENT VISITS AMONG OLDER ADULTS WITH MULTIMORBIDITY

**DOI:** 10.1093/geroni/igad104.3087

**Published:** 2023-12-21

**Authors:** Arum Lim, Chitchanok Benjasirisan, Xiaoyue Liu, Oluwabunmi Ogungbe, Cheryl Dennison Himmelfarb, Patricia Davidson, Binu Koirala

**Affiliations:** Johns Hopkins University, Baltimore, Maryland, United States; Johns Hopkins University, Baltimore, Maryland, United States; Johns Hopkins University, Baltimore, Maryland, United States; Johns Hopkins University, Baltimore, Maryland, United States; Johns Hopkins University, Baltimore, Maryland, United States; University of Wollongong, Wollongong, New South Wales, Australia; Johns Hopkins University School of Nursing, Baltimore, Maryland, United States

## Abstract

Multimorbidity is prevalent in older adults and is related to various adverse health outcomes, including high emergency department (ED) visits. Evidence suggests that social determinants of health (SDoH) are associated with many health outcomes, but the association between SDoH and ED visits among older adults with multimorbidity has received limited attention. This study aimed to examine the association between SDoH and ED visits among older adults with multimorbidity. A cross-sectional analysis was conducted among 25,078 adults aged 50 years and older from the 2010 to 2018 National Health Interview Survey. Multimorbidity was defined as the presence of two or more self-reported diseases among nine common chronic conditions, such as hypertension, diabetes, asthma, stroke, and cancer. The SDoH assessed included race, education, poverty, marital status, employment status, insurance status, and having a usual place for medical care. Participants’ mean (±SD) age was 68.06 (±10.16) years, and 55.13% were female. After adjusting for age, sex, and categories of comorbidities in the logistic regression model, high school or less education (AOR=1.14, 95%CI=1.05-1.24), poverty income ratio < 1 (AOR=1.50, 95%CI=1.35-1.66), unmarried (AOR=1.23, 95%CI=1.15-1.32), and unemployment status (AOR=1.36, 95%CI=1.25-1.48) had higher odds of having 1 or more ED visits. Non-Hispanic Blacks had higher odds (AOR=1.28, 95%CI=1.18-1.40), while non-Hispanic Asians had lower odds (AOR=0.69, 95% CI=0.56-0.84) of 1 or more ED visits than non-Hispanic Whites. SDoH are associated with ED visits among older adults with multimorbidity; providers should consider SDoH in health-seeking behaviors and to promote health equity.

